# Supplementary material for: The role of the strain induced population imbalance in Valley polarization of graphene: Berry curvature perspective
Source: Sci Rep. 2017 Dec 19;7:17878. doi: 10.1038/s41598-017-18238-5 (PMC5736734; doi:10.1038/s41598-017-18238-5)
Supplement: Supplementary file 1 — Supplementary Information [file 41598_2017_18238_MOESM1_ESM.pdf]

Supplementary Information
The role of the strain induced population imbalance in Valley polarization of graphene: Berry curvature perspective

Tohid Farajollahpour* and Arash Phirouznia
Department of Physics, Azarbaijan Shahid Madani University, 53714-161, Tabriz, Iran
Condensed Matter Computational Research Lab. Azarbaijan Shahid Madani University, 53714-161, Tabriz, Iran

I. GAUGE FIELDS

In this section of supplementary information we introduce two gauge fields that have been considered in the present study. Different perturbation mechanisms in Dirac equation, could be described by an effective gauge field mathematically. In the Hamiltonian of the honeycomb lattice addition of a gauge field corresponds to change of the hopping between the sub-lattices that reveals in the off diagonal elements of the Dirac equation. In the current work we have considered two different types of gauge fields: Real magnetic gauge which describes the presence of an external magnetic field [1], pseudo-magnetic gauge field that originates from lattice deformation (fictitious gauge field) [2].

A. REAL MAGNETIC FIELD

External real magnetic field alone in graphene can not lift the valley degeneracy, while it breaks the time-reversal symmetry of the Hamiltonian [3]. The effect of vector potential of an external magnetic field on the tight-binding Hamiltonian of a honeycomb lattice can be obtain by Pierels substitution. It corresponds to change the hopping matrix element t_{ij} to $t_{ij}e^{i\int_j^i A(r).d\boldsymbol{r}}$, in which $A(r)$ is the vector potential and the selected real magnetic gauge fields are:

$$A_M = \frac{r \times B}{2} \tag{1.1}$$

where r indicate the electron position, B denotes the external magnetic field. t_{ij} is the nearest neighbor hopping term so the real space Peierl's substitution in the Dirac Hamiltonian of graphene reads

$$H_D^{(M)} = H_{gr} + H_M \tag{1.2}$$

where

$$H_{gr} = \int d^2r \hat{\Psi}^{\dagger}(r) \upsilon_f (\boldsymbol{\gamma}\cdot(-\boldsymbol{i}\boldsymbol{\nabla}))\hat{\Psi}(r) \tag{1.3}$$

and

$$H_M = \int d^2r \hat{\Psi}^{\dagger}(r) \upsilon_f \boldsymbol{\gamma}\cdot(e\boldsymbol{A}_M)\hat{\Psi}(r), \tag{1.4}$$

in which $\hbar = c = 1$ and the four component spinor operator is

$$\hat{\Psi}(r) = \begin{pmatrix} \hat{\Psi}_{+A}(r) \\ \hat{\Psi}_{+B}(r) \\ \hat{\Psi}_{-B}(r) \\ \hat{\Psi}_{-A}(r) \end{pmatrix}, \tag{1.5}$$

which obeys the fermion algebra and υ_f is the Fermi velocity in graphene. The index $\pm$ describes two inequivalent Dirac points at the corners of the graphene Brillouin zone. The A and B indexes indicate two different real space sub-lattices of the

*Electronic address: Tohidfrjpr@gmail.com

honeycomb structure. In the above representation of Hamiltonian, the choice of the four-dimensional chiral representation of Dirac matrices are,

$$\boldsymbol{\gamma} := \begin{pmatrix} \boldsymbol{\tau} & 0 \\ 0 & -\boldsymbol{\tau} \end{pmatrix} \equiv \sigma_3 \otimes \boldsymbol{\tau} \equiv \left(\gamma^1, \gamma^2\right)$$
$$\gamma^3 := \begin{pmatrix} \tau_3 & 0 \\ 0 & -\tau_3 \end{pmatrix} \equiv \sigma_3 \otimes \tau_3 \tag{1.6}$$

The three Pauli matrices (τ_1, τ_2 and τ_3) and 2×2 unit matrix τ_0 act on the sub-lattice indices (A and B), while the Pauli matrices that operate on the valley indices ($\pm$) are presented by the σ_1, σ_2 and σ_3.

B. GAUGE FIELD FROM LATTICE DEFORMATION

Because of the strong pseudomagnetic field which induced by strain in graphene, the strain engineering of the graphene has become an alternative tool for manipulating the energy dispersion in electronic based applications [2, 4–8]. It has been proven that the strain cannot break the time-reversal symmetry. Since the strain acts oppositely at different valleys of graphene[3]. The elastic deformations in honeycomb lattice have been considered as gauge fields in graphene Hamiltonian. At finite temperatures because of the thermal fluctuations these types of deformations and related gauge fields are intrinsic [9]. The distance between the real space lattice sites, is modified as a result of the lattice deformation. The gauge fields for small displacement $\vec{u}\,(\vec{r})$ are obtained as [2, 10–12] meanwhile, the limitation of following equations has been discussed in detail in Ref. [12].

$$A_{S_x} = \frac{\beta t}{e v_f}\left(u_{xx} - u_{yy}\right)$$
$$A_{S_y} = \frac{-2\beta t}{e v_f} u_{xy}, \tag{1.7}$$

$\beta = -\frac{\partial \ln t}{\partial \ln a} \simeq 2$ is the Gruneisen parameter and $u_{\alpha\beta} = \frac{1}{2}(\partial_\alpha u_\beta + \partial_\beta u_\alpha)$. The remarkable point in these fields is their dependence to the microscopic details of the materials[2]. It should be mentioned that strains can generate off diagonal terms in Dirac Hamiltonian and for simplification of the analysis we choose an especial type of strain that the scalar potential $u_{xx} + u_{yy}$ has to be eliminated [10, 11]. Because of time reversal invariance of the strain induced gauge, the corresponding vector potential is oppositely directed at different valleys. The Dirac Hamiltonian of the graphene in the presence of fictitious gauge field is,

$$H_D^{(S)} = H_{gr} + H_S \tag{1.8}$$

where

$$H_S = \int d^2 r \hat{\Psi}^\dagger(r) v_f \gamma^5 (\boldsymbol{\gamma} \cdot \boldsymbol{A}_S) \hat{\Psi}(r), \tag{1.9}$$

in which

$$\gamma^5 := -i\gamma^1\gamma^2\gamma^3 = \begin{pmatrix} \tau_3 & 0 \\ 0 & -\tau_3 \end{pmatrix} \equiv \sigma_3 \otimes \tau_0. \tag{1.10}$$

The direction of the applied strain relative to the real space orientation of graphene, identified by the zigzag and armchair directions, has been chosen in a manner, which is not difficult to realize experimentally[13]. In the present work, the sample coordinates are assumed so that the y axis corresponds to armchair direction. One possible function for an uniform pseudomagnetic field which is given by the atomic displacements considered in Ref. 4 are expressed as

$$u_x = -K_s(2x_a y_a - y_a^2)$$
$$u_y = K_s(x_a^2 + y_a^2) \tag{1.11}$$

in Cartesian coordinates in which x_a and y_a denote the atomic position. In this modified triaxial strain the variable K_s is the geometrical parameter. The fictitious gauge field that could be obtained by these assumptions are as follows,

$$A_{S_x} = \frac{-4\beta t K_s y_a}{e v_f}$$
$$A_{S_y} = \frac{-2\beta t K_s y_a}{e v_f}. \tag{1.12}$$

The pseudo magnetic field B^{ps} under special case of non-uniform strain is,

$$B^{ps} = \nabla \times A_S = \frac{4\beta t K_s}{e v_f} \tag{1.13}$$

in which for $\beta = 2$, $t = 2.7$, $K_s = 0.5\mu m^{-1}$ and $v_f \simeq 10^6$ generated pseudo magnetic field is $B^{ps} \simeq 10.8\,T$.

II. GEOMETRICAL APPROACH - BERRY CURVATURE

The Berry phase γ_n, for adiabatic transport in parametric R-space around a closed curve C is defined as,

$$\gamma_n = \oint_C d\boldsymbol{R} \cdot \boldsymbol{A}_n(\boldsymbol{R}) \tag{2.1}$$

where

$$\boldsymbol{A}_n(\boldsymbol{R}) = i \langle n(\boldsymbol{R})| \frac{\partial}{\partial \boldsymbol{R}} |n(\boldsymbol{R})\rangle \tag{2.2}$$

and

$$\boldsymbol{\Omega}_n(\boldsymbol{R}) = \boldsymbol{\nabla}_{\boldsymbol{R}} \times \boldsymbol{A}_n(\boldsymbol{R}), \tag{2.3}$$

in which the vector $\boldsymbol{A}_n(\boldsymbol{R})$ is Berry connection and $\boldsymbol{\Omega}_n(\boldsymbol{R})$ is the Berry curvature in the given parametric space. The Berry connection is gauge dependent, whereas the Berry curvature is a gauge invariant quantity. The Berry curvature vanishes when the system is invariant under the time reversal and spatial inversion symmetries. k-space Berry curvature, Ω_{kk}, measures the velocity corrections of Bloch electrons. Here in our case the Hamiltonian of the system is like a two level system Hamiltonian. For a general two level system as given by the following Hamiltonian

$$H = h(R) \cdot \sigma \tag{2.4}$$

where σ is Pauli matrices and $h(R)$ depends on a set of parameters R, then the Berry curvature of the system could be defined as [14],

$$\Omega_{R_1,R_2} = \frac{1}{2} \frac{\partial(\varphi, \cos\theta)}{\partial(R_1, R_2)} \tag{2.5}$$

In spherical coordinates of the parametric space identified by R_1, R_2, R_3 (see Fig. 1) one can define,

$$tan(\varphi) = \frac{R_2}{R_1} \Rightarrow \frac{\partial \varphi}{\partial R_1}(1 + tg^2\varphi) = \frac{-R_2}{R_1^2}. \tag{2.6}$$

So

$$\frac{\partial \varphi}{\partial R_1} = \frac{-R_2}{R_1^2 + R_2^2} \tag{2.7}$$

and similarly

$$\frac{\partial \varphi}{\partial R_2} = \frac{R_1}{R_1^2 + R_2^2}, \tag{2.8}$$

in the next step we define $\cos\theta$ in terms of R_1, R_2, R_3 (Fig. 1) and get to following relations,

$$\frac{\partial \cos\theta}{\partial R_1} = \frac{-R_3 R_1}{(R_1^2 + R_2^2 + R_3^2)^{3/2}} \tag{2.9}$$

$$\frac{\partial \cos\theta}{\partial R_2} = \frac{-R_3 R_2}{(R_1^2 + R_2^2 + R_3^2)^{3/2}} \tag{2.10}$$

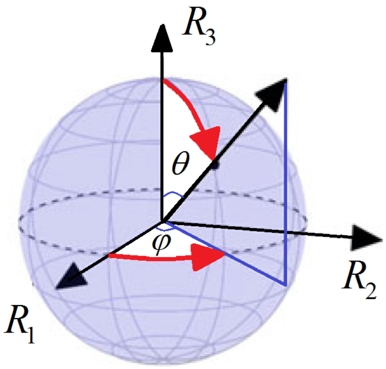

FIG. 1: The spherical coordinates of R_1, R_2, R_3

So by considering the above relations we have,

$$\Omega_{R_1R_2} = \frac{1}{2}\begin{vmatrix} \frac{\partial\varphi}{\partial R_1} & \frac{\partial\cos\theta}{\partial R_1} \\ \frac{\partial\varphi}{\partial R_2} & \frac{\partial\cos\theta}{\partial R_2} \end{vmatrix} \tag{2.11}$$

$$\Omega_{R_1R_2} = \frac{R_3}{2(R_1^2+R_2^2+R_3^2)^{3/2}} \tag{2.12}$$

Then for a Berry vector we will have

$$\mathbf{\Omega} = \frac{1}{2}\frac{\mathbf{h}}{h^3} \tag{2.13}$$

Where $\mathbf{h} = (R_1, R_2, R_3)$. By means of Eq. 2.13, the Berry curvature of each two level system can be calculated.

III. PERTURBATION METHOD

In this appendix first order perturbation method has been applied to treat the influence of the magnetic field on Dirac fermions in strained graphene. The first order energy correction, could be obtained by

$$\mathcal{E}^{\pm}_{\eta,\mathbf{k}} = \mathcal{E}^{(0)\pm}_{\eta,\mathbf{k}} + \sum\langle\psi^{(0)}_{\tau\eta}|\hat{A}_M|\psi^{(0)}_{\tau\eta}\rangle, \tag{3.1}$$

where A_M is the non-momentum transfer part of the magnetic gauge field. For obtaining eigenfunctions and eigenvalues of the strained graphene one has to solve

$$H^{(0)} = \begin{bmatrix} m & P^- & 0 & 0 \\ P^+ & -m & 0 & 0 \\ 0 & 0 & -m & Q^- \\ 0 & 0 & Q^+ & m \end{bmatrix}. \tag{3.2}$$

for each valley point of graphene,

$$\left(\mathcal{H}^{(0)}_\eta - \mathcal{E}^{(0)}_{\eta,\mathbf{k}}\right)\psi^{(0)}_\eta = 0. \tag{3.3}$$

The eigenvalues and eigenvectors are,

$$\mathcal{E}^{(0)\pm}_{\eta,\mathbf{k}} = \pm\sqrt{m^2 + v_f^2(k^+ + \eta A_s^+)(k^- + \eta A_s^-)} \tag{3.4}$$

where valley index η is $+(-)$ for $K(K')$ and

$$\psi^{(0)}_{\tau\eta} = \begin{pmatrix} \alpha_{\tau\eta} \\ \\ \beta_{\tau\eta} \end{pmatrix} \tag{3.5}$$

By above considerations, we can write the perturbation correction for the two Dirac points of honeycomb lattice as follows

$$\Delta\mathcal{E}^s_\eta = \eta e v_f \left(\langle k|A^+_M|k\rangle \alpha_{s\eta}\beta^*_{s\eta} + \langle k|A^-_M|k\rangle \alpha^*_{s\eta}\beta_{s\eta}\right) \tag{3.6}$$

where

$$A_M = \begin{bmatrix} 0 & A^-_M \\ A^+_M & 0 \end{bmatrix}. \tag{3.7}$$

The value of spectral gap around K and K' is therefore given by

$$\Delta\mathcal{E}^g_{K(K')} = \left(\mathcal{E}^{(0)+}_{K(K')} + \Delta\mathcal{E}^+_{K(K')}\right) - \left(\mathcal{E}^{(0)-}_{K(K')} + \Delta\mathcal{E}^-_{K(K')}\right) \tag{3.8}$$

The valleys gap difference is

$$\Delta E^{valleys} = \Delta\mathcal{E}^g_K - \Delta\mathcal{E}^g_{K'}, \tag{3.9}$$

after some simple calculations it is obvious that the first order perturbation could generate valley gap difference is

$$\Delta E^{valleys} = \frac{e v_f B L^2}{2} \sum_\eta \left(\mathcal{M}_{+\eta} - \mathcal{M}_{-\eta}\right) \tag{3.10}$$

where $\mathcal{M}_{+\eta} = \mathcal{A}_{+\eta} + \mathcal{A}^*_{+\eta}$, $\mathcal{M}_{-\eta} = \mathcal{A}_{-\eta} + \mathcal{A}^*_{-\eta}$ and $\mathcal{A}_{s\eta} = \alpha_{s\eta}\beta^*_{s\eta}$. The α and β's are the components of the unperturbed Dirac Hamiltonian eigenvectors. In the case of the K valley point and $E^{(0)+}$, these two terms are given by:

$$\alpha_{+K} = \left(\frac{v_f^2|k^+ + As^+|^2}{v_f^2|k^+ + As^+|^2 + (m + E^{(0)+}_K)^2}\right)^{\frac{1}{2}} \tag{3.11}$$

and

$$\beta_{+K} = \left(\frac{(m + E^{(0)+}_K)^2}{v_f^2|k^+ + As^+|^2 + (m + E^{(0)+}_K)^2}\right)^{\frac{1}{2}}. \tag{3.12}$$

For the same valley and the eigen energy $E^{(0)-}$ we have

$$\alpha_{-K} = -\left(\frac{v_f^2|k^- + As^-|^2}{v_f^2|k^- + As^-|^2 + (m - E^{(0)-}_K)^2}\right)^{\frac{1}{2}} \tag{3.13}$$

and

$$\beta_{-K} = \left(\frac{(m - E^{(0)-}_K)^2}{v_f^2|k^- + As^-|^2 + (m - E^{(0)-}_K)^2}\right)^{\frac{1}{2}}. \tag{3.14}$$

In the other valley K' with $E^{(0)+}$

$$\alpha_{+K'} = \left(\frac{v_f^2|k^- - As^-|^2}{v_f^2|k^- - As^-|^2 + (m + E^{(0)+}_{K'})^2}\right)^{\frac{1}{2}} \tag{3.15}$$

and

$$\beta_{+K'} = \left(\frac{(m + E^{(0)+}_{K'})^2}{v_f^2|k^- - As^-|^2 + (m + E^{(0)+}_{K'})^2}\right)^{\frac{1}{2}}. \tag{3.16}$$

finally for E^{0-} in valley K' it can be easily shown that

$$\alpha_{-K'} = -\left(\frac{v_f^2|k^+ - As^+|^2}{v_f^2|k^+ - As^+|^2 + (m - E^{(0)-}_{K'})^2}\right)^{\frac{1}{2}} \tag{3.17}$$

and

$$\beta_{-K'} = \left(\frac{(m - E_{K'}^{(0)-})^2}{v_f^2 |k^+ - As^+|^2 + (m - E_{K'}^{(0)-})^2} \right)^{\frac{1}{2}}. \tag{3.18}$$

[1] M. Goerbig, Reviews of Modern Physics **83**, 1193 (2011).
[2] M. A. Vozmediano, M. Katsnelson, and F. Guinea, Physics Reports **496**, 109 (2010).
[3] K.-J. Kim, Y. M. Blanter, and K.-H. Ahn, Physical Review B **84**, 081401 (2011).
[4] A. Vaezi, N. Abedpour, R. Asgari, A. Cortijo, and M. A. Vozmediano, Physical Review B **88**, 125406 (2013).
[5] E. Arias, A. R. Hernández, and C. Lewenkopf, Physical Review B **92**, 245110 (2015).
[6] S. Zhu, J. A. Stroscio, and T. Li, Physical review letters **115**, 245501 (2015).
[7] N. N. Klimov, S. Jung, S. Zhu, T. Li, C. A. Wright, S. D. Solares, D. B. Newell, N. B. Zhitenev, and J. A. Stroscio, Science **336**, 1557 (2012).
[8] N. Levy, S. Burke, K. Meaker, M. Panlasigui, A. Zettl, F. Guinea, A. C. Neto, and M. Crommie, Science **329**, 544 (2010).
[9] A. Fasolino, J. Los, and M. I. Katsnelson, Nature materials **6**, 858 (2007).
[10] H. Suzuura and T. Ando, Physical review B **65**, 235412 (2002).
[11] J. L. Manes, Physical Review B **76**, 045430 (2007).
[12] M. R. Masir, D. Moldovan, and F. Peeters, Solid State Communications **175**, 76 (2013).
[13] F. Guinea, M. Katsnelson, and A. Geim, Nature Physics **6**, 30 (2010).
[14] D. Xiao, M.-C. Chang, and Q. Niu, Reviews of modern physics **82**, 1959 (2010).
